# Airbag injuries of the hand: a case-series and literature review

**DOI:** 10.1093/jscr/rjae052

**Published:** 2024-02-13

**Authors:** Asmaa Z B Mahmoud, Abdullah M Alhusain, Mohammed N H Alkhadhrawi, Dana C Vasilescu

**Affiliations:** Plastic and Reconstructive Surgery Division, Surgery Department, Ministry of National Guards Health Affairs, King Abdulaziz Medical City, P.O. Box 22490, Riyadh 14611, Saudi Arabia, Saudi Arabia; Plastic and Reconstructive Surgery Division, Surgery Department, Ministry of National Guards Health Affairs, King Abdulaziz Medical City, P.O. Box 22490, Riyadh 14611, Saudi Arabia, Saudi Arabia; King Saud Bin Abdulaziz University for Health Sciences, P.O. Box 3660, Riyadh 11481, Saudi Arabia; Plastic and Reconstructive Surgery Division, Surgery Department, Ministry of National Guards Health Affairs, King Abdulaziz Medical City, P.O. Box 22490, Riyadh 14611, Saudi Arabia, Saudi Arabia

**Keywords:** airbag, upper extremity, hand, injury, motor vehicle accident

## Abstract

Motor vehicle accidents are a significant cause of morbidity and mortality worldwide. Airbags aim to reduce the severity of motor vehicle accidents, but their deployment is not without risks. This study presents five cases presenting with diverse forms of upper extremity injuries following airbag deployment. The presented cases highlight the variety of clinical presentations, the differences in diagnostics in terms of imaging modalities, as well as the spectrum of possible outcomes from complete healing to decreased range of motion to persistent neurological symptoms. Understanding the mechanisms and presentations of such injuries can only help in improving and creating new strategies for the prevention of such injuries, as well as their management.

## Introduction

Motor vehicle accidents (MVAs) are a major cause of mortality, as well as physical and psychological morbidity [1]. The Centers for Disease Control and Prevention (CDC) published a report examining deaths caused by MVAs between 2015 and 2019 in 29 high-income countries. The results showed that there are close to 2500 deaths on average every year [2]. Therefore, automobile manufacturers began installing safety equipment such as airbags into their vehicles to curtail these adverse events.

Despite their protective purposes, numerous reports in the literature indicate that they are a potential source of harm. According to some author’s estimates, ~43% of airbag deployments result in one or more injuries [3]. The upper limb was reported as either the first [4] or second most commonly affected body part in injuries caused by airbags [5, 6]. The occurrence of injury to the upper limb was up to four times more likely in MVAs with airbag deployment compared to those without [4, 7]. Thus, we present the following cases.

## Case 1

**Figure 1:**
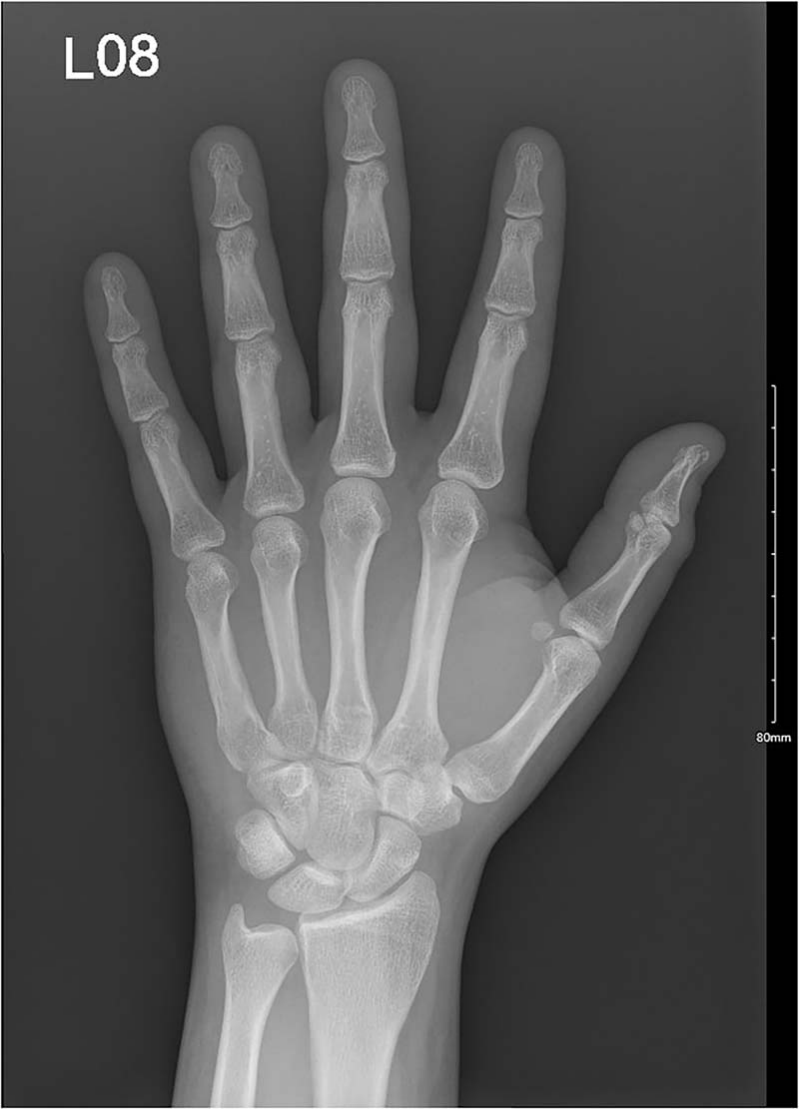
A comminuted slightly displaced fracture of the tip of the left fifth distal phalanx.

**Figure 2:**
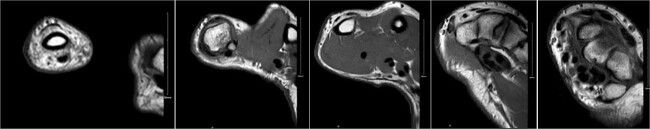
Complete tear of ulnar collateral ligament with significant proximal retraction.

A 32-year-old male presented to the emergency department with a painful, discolored, and edematous left thumb following spontaneous deployment of his automobile’s airbag. On physical examination, the thumb’s range of motion (ROM) was reduced because of pain. Radiography showed a tuft fracture of the thumb’s distal phalanx (Fig. 1). The patient was managed conservatively with a Zimmer splint and followed-up 1 week later. Magnetic resonance imaging (MRI) was obtained to assess for the presence of ligamentous injury showed complete tearing and proximal retraction of the distal attachment of the ulnar collateral ligament in (Fig. 2), so the patient was given a thumb spica and was booked for surgery. In the operating room, the ulnar collateral ligament was identified and was indeed completely avulsed from its distal attachment site. The ligament was repaired using the Kessler tendon repair maneuver, ultimately ending with the thumb flexed 30° at the metacarpophalangeal (MCP) joint. This patient was followed-up for 103 days after his first presentation. On his final clinic visit, the thumb was stable, the wound was fully healed, and full ROM was regained.

## Case 2

A 30-year-old female was involved in a motor vehicle accident as an unrestrained passenger. When the airbags got deployed, the patient attempted to shield her face with her hands, leading to injury to her right fifth finger. On examination, the finger was painful, swollen, bruised, and deformed. The digit was very tender and had a limited ROM. A hand X-ray was acquired, and it showed an avulsion fracture and dorsal dislocation of the middle phalanx and its base, respectively. Attempted reduction in the emergency department had failed, so the patient was admitted for surgery. In the operating room (OR), closed reduction and internal fixation with a k-wire was performed, and proper alignment was confirmed to be successful by fluoroscopy. Initially, a volar splint had been applied to the patient, but it was changed to an ulnar gutter splint 1 week after the surgery. The patient reported tenderness at the metacarpophalangeal joint when she was last seen at the clinic.

## Case 3

**Figure 3:**
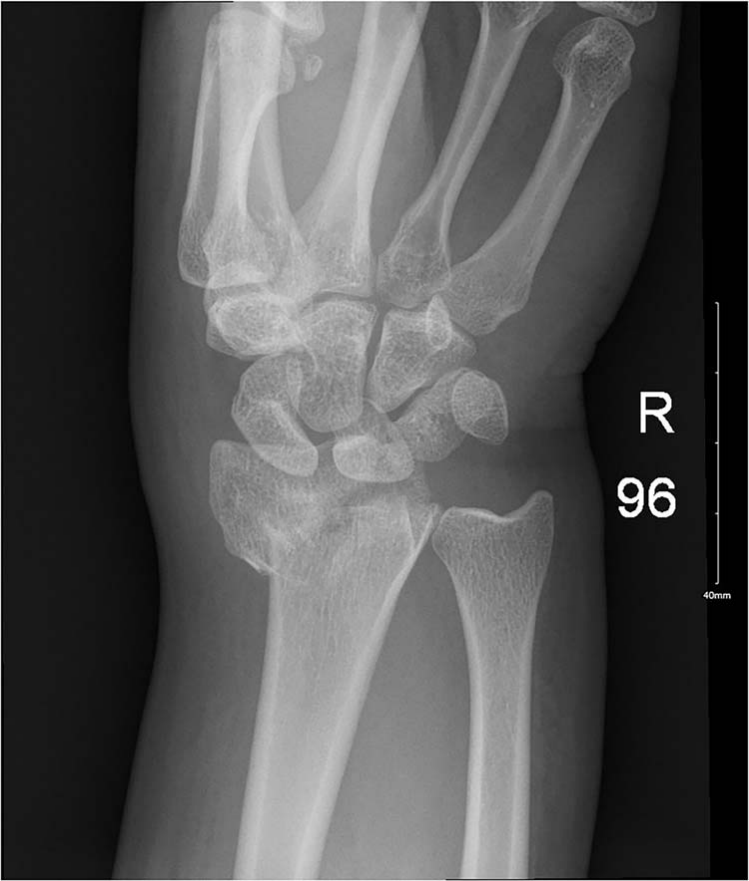
Impacted comminuted fracture of the distal radius.

**Figure 4:**
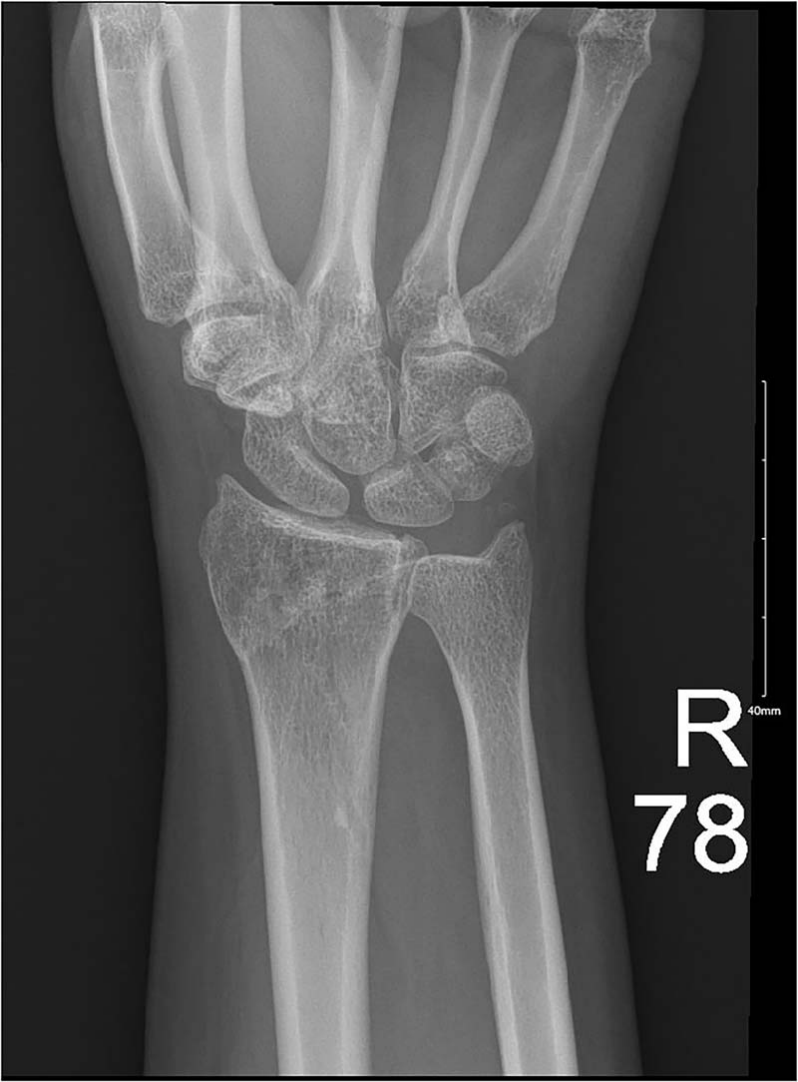
Ongoing healing is seen in the distal radius fracture. The fracture line is barely visible.

A 29-year-old female presented to the emergency department with pain and deformity of her right wrist as well as a laceration of her right thumb following an MVA with airbag deployment as an unrestrained passenger. The incident happened when another vehicle crashed sideways into the driver’s side while moving at ~50 kmph. There was limited ROM at the MCP joint due to pain. Imaging showed an oblique fracture at the base of first metacarpal bone medially (Fig. 3), so the patient was admitted for wound exploration and repair of other concomitant injuries. The wound was irrigated, the neurovasculature and tendons were observed to be intact, the laceration was sutured, and thumb spica was applied. Subsequent clinic visits showed a hyperpigmented scar as well as a decreased ROM in her wrist and fingers as the radius was still going through the healing process (Fig. 4).

## Case 4

A 25-year-old female presented with a laceration on her right hand following an MVA with airbag deployment as an unrestrained front passenger. On examination, there was a 12 cm deep laceration on the 1st web space, extending from the dorsum of the hand, crossing the web space, and reaching to end of thenar eminence with visible severed muscles. There was also a 2–3 cm deep laceration over 4th web space extending from the distal palmar crease crossing the web and reaching the dorsum of web space. Assessment of finger movement was normal except for loss of thumb adduction. Wrist X-ray and computed tomography (CT) showed a non-displaced fracture at the dorsum of the triquetrum as well as a 4.8 × 2.6 cm subcutaneous/intramuscular hematoma overlying the thenar area associated with multiple soft tissue emphysema. In the OR, the superficial and deep heads of the adductor pollicis muscle, as well as the dorsal sensory branch going to the index finger were found to be completely cut. Both the nerve and muscle were sutured, and a thumb spica was applied. In the patient’s last clinic visit, the wound was healed and full ROM was regained, but the patient developed a positive Tinel sign.

## Case 5

**Figure 5:**
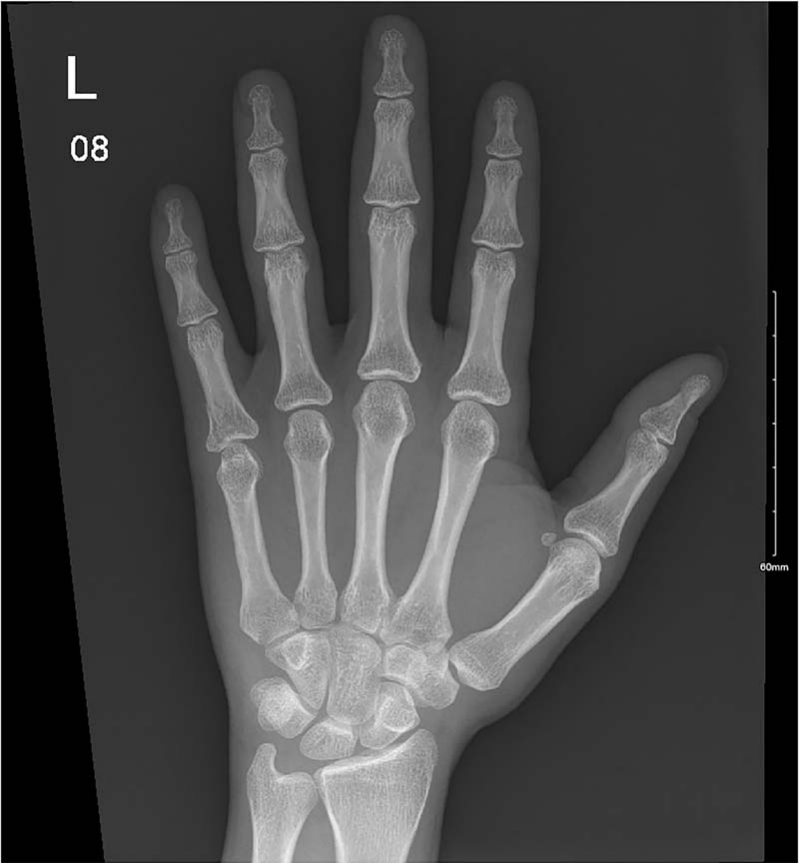
A minimally displaced fracture at the base of proximal fifth phalanx extending into the articular surface.

**Figure 6:**
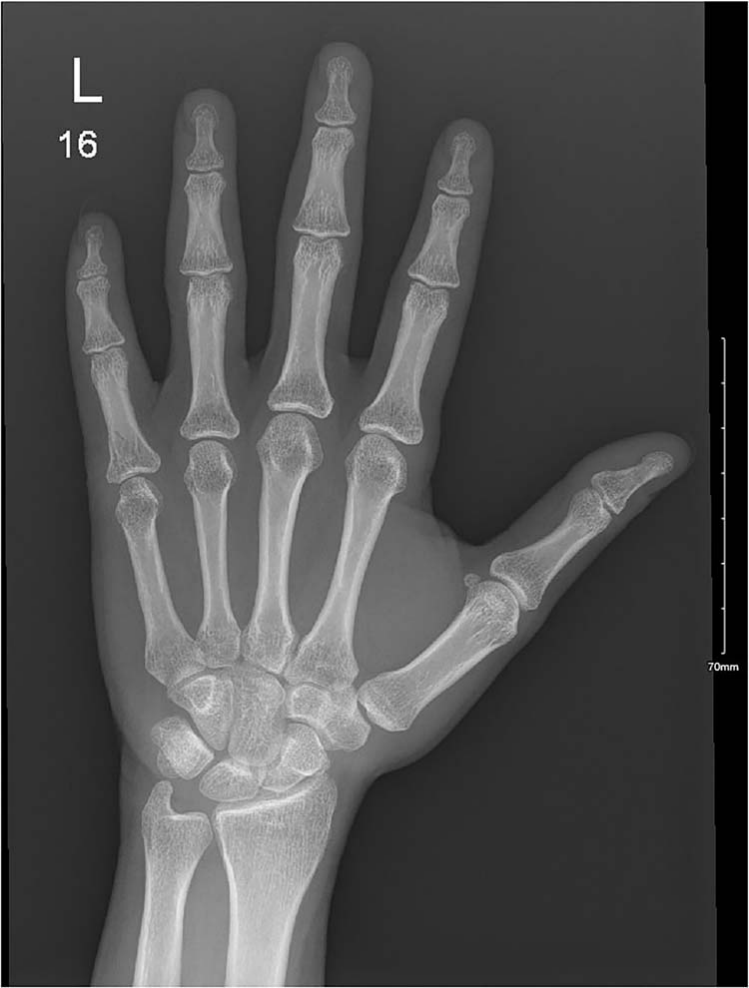
Fracture at the fifth proximal phalanx with ongoing healing.

A 28-year-old female came to the emergency room (ER) following an MVA with airbag-deployment 5 days before presentation. Examination revealed tenderness at the base of the proximal fifth finger. The ROM of the affected digit was limited, but the neurovasculature as well as flexor digitorum profundus and flexor digitorum superficialis function were intact. X-ray of the hand showed a minimally displaced fracture at the base of the proximal fifth phalanx extending into the articular surface (Fig. 5). The decision was made to manage the patient conservatively by applying an ulnar gutter splint, which was then changed to a volar splint when the patient was followed-up in the clinic a week later. When the patient was last seen, the patient had limited ROM, and the tenderness had resolved. Imaging showed evidence of ongoing healing (Fig. 6).

## Discussion

Airbags are pivotal devices designed to mitigate the severity of injuries sustained during MVAs. During a crash, there are three main collisions that can happen. The first is between the vehicle and another object. The second is between those inside the vehicle and the vehicle’s interior. And the third is between the organs of the vehicle’s occupants and the encasing body wall or cavity [8]. While their primary goal is to protect occupants from colliding with the vehicle's interior, the deployment of airbags is not without consequences, particularly for the upper extremities. Understanding the mechanisms and patterns of airbag-induced injuries to the upper limb is crucial for developing effective preventive strategies and especially optimizing post-accident management in the short and long terms.

The deployment of an airbag is a rapid and forceful event which is triggered by sensors, usually in the vehicle’s bumpers, detecting abrupt deceleration during impact. This inflation process generates temperatures reaching up to 700°C in fractions of a second, initiating a cascade of chemical reactions ultimately ending with the release of heat, nitrogen, and many more chemicals which are all a potential cause of injury [8]. The forces exerted by the inflating airbag can lead to various injuries in the upper extremities in spite of their protective purpose. The literature reports that injuries often occur when the arm is caught between the rapidly inflating airbag and the torso, when the limb is forcefully displaced during deployment, or when the hand is stuck in the spokes of the steering wheel [5, 9].

Studies conducted in the United States have documented a significant number of injuries attributed to airbags, with the face, wrist, and forearm emerging as the most commonly affected areas [5]. Notably, research by Richter *et al.* [10] and further supported by Chong *et al.* [11] underscores the vulnerability of the hand and wrist in restrained vehicle occupants involved in car crashes. However, Freedman's findings differ slightly, emphasizing the susceptibility of the first and fifth digits over more proximal injuries [12]. The spectrum of reported injuries encompasses abrasions, contusions, fractures, and burns [9, 13–16], with the majority of injuries affecting soft tissues rather than bones [11]. In a comprehensive study by Jernigan *et al.*, which examined over 25 000 car crashes resulting in upper extremity injuries, ~19% of cases involved fractures of the hand [7].

The cases reported above demonstrate the variability in presentations, and the diverse range of injuries that can result from airbag deployment including ligamentous avulsions, fractures, and complex soft tissue injuries. Further, challenges to the management were illustrated. While some patients achieved full recovery (Case 1), others experienced lingering issues such as decreased ROM (Case 3, Case 5) and the development of a positive Tinel sign (Case 4).

In conclusion, airbag deployment in MVAs presents a notable risk for upper extremity injuries. The hand and digits, in particular, emerge as one of the most frequently affected regions. Building a thorough understanding of the mechanisms leading to such injuries is essential for developing effective strategies for the prevention of such injuries, as well as their management in the short and long term.

## Conflict of interest statement

None declared.

## Funding

None declared.
